# Clinical Performance of Lateral Flow Assay for *Cryptosporidium* spp. Diagnosis

**DOI:** 10.3390/biomedicines11082140

**Published:** 2023-07-29

**Authors:** Miriam Campos-Ruiz, Clara Flamarich, Anabel Fernández-Navarro, Silvia Roure, Laura Martin, Pablo Pillado, Pere-Joan Cardona, Gema Fernández-Rivas

**Affiliations:** 1 Microbiology Department, Clinical Laboratory North Metropolitan Area, Germans Trias i Pujol University Hospital, 08916 Badalona, Spain; mcamposruiz.germanstrias@gencat.cat (M.C.-R.); afernandezn.ics@gencat.cat (A.F.-N.); ppilladoa.germanstrias@gencat.cat (P.P.); pcardonai.germanstrias@gencat.cat (P.-J.C.); 2 Department of Genetics and Microbiology, Autonomous University of Barcelona, 08916 Badalona, Spain; 3 CAP Sant Roc. Catalan Institut of Health, 08916 Badalona, Spain; cflamaricg.mn.ics@gencat.cat; 4 North Metropolitan International Health Program (PROSICS), 08916 Badalona, Spain; sroura.mn.ics@gencat.cat; 5 Infectious Diseases Department, Germans Trias i Pujol University Hospital, Universitat Autònoma de Barcelona, 08196 Badalona, Spain; 6 CAP Doctor Robert Catalan Institut of Health, 08915 Badalona, Spain; lmartinc.ics@gencat.cat

**Keywords:** *Cryptosporidium* spp., epidemiology, lateral flow, antigen detection

## Abstract

*Cryptosporidium* spp. is an apicomplexan protozoan parasite associated with gastroenteritis in humans. In 2018, Spain showed 1511 confirmed cases, with a growing trend since 2014. Despite this fact, *Cryptosporidium* spp. is not usually routinely examined when a parasitological study is ordered, although accurate diagnosis is fundamental to prevent the spread of the illness. The main objectives of the present work is to demonstrate the circulation and to study the epidemiology of cryptosporidiosis in patients who were being tested for the presence of *Cryptosporidium* spp. parasites in the faeces in the Metropolitan North Area of Barcelona, Maresme, and Vallés Occidental using a two-step algorithm. The stool samples were analysed using the *Cryptosporidium*/*Giardia* spp. immunochromatographic test; the positive samples were visualised under a microscope using auramine staining. The proportion of *Cryptosporidium* spp. cases was around 2% in the studied patients, with a pronounced seasonal incidence peak in late summer–early autumn. In our cohort, weight loss was the main symptom related to confirmed cases. The mean age of confirmed patients was 19 years old, and they were younger than the unconfirmed group. *Cryptosporidium* spp. is one of the parasites that currently circulate in many areas in Europe. Prevalence must be taken into account for active searching.

## 1. Introduction

*Cryptosporidium* spp. is an apicomplexan protozoan parasite that can cause gastroenteritis in a variety of vertebrate hosts, including animals and humans [1]. As with other apicomplexan parasites, its life cycle is complicated, alternating between asexual and sexual reproduction. Sporulated oocysts are excreted through the faeces of infected subjects. Following ingestion, excystation occurs, and the sporozoites parasitise epithelial cells of the intestinal tract. In these cells, the parasites undergo schizogony or merogony and then gametogony or sexual multiplication, producing microgamonts and macrogamonts [2]. Upon fertilisation, two types of oocysts develop and sporulate in the infected host. Thin-walled oocysts participate in the autoinfective cycle, whereas thick-walled oocysts are excreted into the environment, boosting the spread [2]. Direct transmission occurs through the faecal–oral route from the infected host (animals or humans) [3]. Indirect transmission involves contact with material contaminated with *Cryptosporidium* spp. oocysts such as food, water, clothes, and footwear [3]. Furthermore, it is also possible to inhale *Cryptosporidium* spp. oocysts, especially described in children and immunocompromised patients [4]. One of the main factors that contribute to *Cryptosporidium* species oocysts spreading is that they can survive in a wide range of environments at ambient temperatures for many months. Additionally, an infection caused by *Cryptosporidium* species requires only a few oocysts, as their infectious dose is low. Furthermore, these oocysts are resistant to common treatments of drinking water, such as chlorine [5].

Currently, *Cryptosporidium* spp. and *Giardia* spp. are the most commonly reported etiological agents of waterborne outbreaks caused by parasites worldwide [6]. Additionally, in a 2015 global burden disease mortality review, cryptosporidiosis was one of the three most common diarrhoea etiologies in children (among rotavirus and *Shigella* spp.), causing 60,400 deaths [7]. Although at least 39 species of *Cryptosporidium* spp. have been described, specific subtypes of the zoonotic *Cryptosporidium parvum* and the anthroponotic *Cryptosporidium hominis* are responsible for the majority of human cases in Europe [3]. Interestingly, any relationship has been observed between *Cryptosporidium* species and the clinical manifestations of the infected patients. Cryptosporidiosis is characterised by watery diarrhoea that lasts longer than gastroenteritis caused by viruses and bacteria. It may sometimes be profuse and prolonged, and it can be accompanied by nausea, vomiting, and low-grade fever, although cryptosporidiosis clinical presentation is not specific to this infection. Nevertheless, these symptoms can cause some patients to seek medical attention. In humans, the severity of manifestations can vary from an asymptomatic shedding of the oocysts to a severe and life-threatening illness, especially in immunocompromised patients [8]. Very young children and people with underlying conditions such as HIV/AIDS, cancer, immune-suppressing medication, or malnutrition are at higher risk of developing debilitating diseases and chronic infections and have higher mortality rates. [9,10]. This is one of the reasons why in the 1980s, cryptosporidiosis was included in AIDS-defining illnesses [8]. Cryptosporidiosis is usually self-limiting in immunocompetent patients, but sometimes they can suffer persistent symptoms beyond acute disease, which could be indicative of postinfectious irritable bowel disease [11].

The global incidence of cryptosporidiosis is uncertain. It is estimated that 748,000 cases occur every year in the United States [12]. According to the Surveillance Atlas of Infectious Diseases data from the European Centre for Disease Prevention and Control (ECDC), there were 14,252 reported cases in 2018 in Europe [13]. In Spain, cryptosporidiosis was not subjected to mandatory surveillance and notification until 2009 [14]. Until then, available data related to *Cryptosporidium* spp. epidemiology were limited. Likewise, the ECDC published in its Annual Epidemiology Report that in 2018 Spain had 1511 confirmed cases, with a growing trend since 2014, when 326 cases were reported. This report also showed that Germany, The Netherlands, Spain, and the UK accounted for 76% of all confirmed cases [13]. Although in some European countries, cryptosporidiosis is still not considered a notifiable disease, outbreaks have been described in Sweden, the United Kingdom, and also in Spain [13]. Despite this fact, tests for *Cryptosporidium* spp. are not routinely performed in most laboratories when a parasitological study is ordered; therefore, healthcare providers should specifically request testing for this parasite.

Cryptosporidiosis is underdiagnosed and underreported due to a lack of suspicion, together with the unavailability of diagnostic tests. In fact, currently, there are no international standard methods for the diagnosis of cryptosporidiosis. Conventional faeces culture is difficult, and it is not routinely performed because it requires an in vitro protozoan culture. Serological tests can be used but only for epidemiological interest, as they do not imply an active infection [15]. Although molecular diagnosis options are increasing, faeces microscopy examination remains the gold standard for *Cryptosporidium* spp. infection, as other invasive gastrointestinal samples are more difficult to obtain and are not always suitable (bowel aspiration, for example) [15]. To facilitate cryptosporidiosis surveillance and diagnosis, lateral flow assays (LFIA) are available. These tests are quick test assays with high throughput and are often used in European laboratories [15,16]. In our country, different commercial assays are available and are being increasingly used for diagnosis thanks to the low technical expertise required and short turnaround time. However, LFIA techniques have sensitivity and specificity problems with species other than *C. parvum* and *C. hominis*. As Checkley et al. stated [15], auramine-rhodamine fluorescent stain improves the traditional acid-fast staining techniques due to the improved sensitivity of the fluorescent microscopy. These fluorescent stain methodologies are often used as a gold standard diagnosis technique in reference laboratories in Europe and the United States of America. In high-throughput microbiology laboratories, a screening method could decrease the workload since performing auramine staining on all samples is not a cost-effective strategy. An accurate laboratory diagnosis of cryptosporidiosis is helpful and fundamental for appropriate management of patients, identification of outbreaks, assessment of risk factors, and performing public health interventions. In this line, the main objective of the present work is to demonstrate the circulation and to study the epidemiology of cryptosporidiosis in patients who were being tested for the presence of *Cryptosporidium* spp. parasites in the faeces in the Metropolitan North Area of Barcelona, Maresme, and Vallés Occidental using a two-step algorithm.

## 2. Materials and Methods

A retrospective study of cryptosporidiosis cases diagnosed in the Microbiology and Parasitology service of the Hospital Universitari Germans Trias i Pujol (HUGTiP) was conducted from June 2016 to March 2019.

### 2.1. Antigen Detection of Cryptosporidium spp. Oocysts in Faeces

All stool samples in which parasitological tests were ordered after a medical consultation were analysed using the *Cryptosporidium*/*Giardia* RIDA^®^QUICK immunochromatographic test (ICT) (RIDA^®^QUICK *Cryptosporidium*/*Giardia* Combi, R-Biopharm, Darmstadt, Germany). This screening method allows the rapid qualitative determination of *Cryptosporidium* spp. and *Giardia lamblia* in stool samples. Positive samples were confirmed through the visualisation of oocysts under a microscope (gold standard method). For the Ova and Parasite (O&P) wet mount examination, samples were fixed in a formalin-free 60 fixative, AlcorFix^®^, and Mini Parasep^®^ SF (solvent-free) collection tube (Parasep^®^; Apacor, Berkshire, England, UK) for every positive sample.

### 2.2. Observation of Cryptosporidium spp. Oocyst in Faeces

Faeces positive for *Cryptosporidium* spp. were subsequently fixed with the Mini Parasep^®^ SF (solvent-free) collection tube and were filtered by centrifugation at 1500 rpm for 2 min according to the manufacturer’s instructions. Then, the sediment was smeared onto a glass slide and air-dried. After that, they were stained using auramine Aerospray^®^ TB (Elitech Group, Pouteaux, France). Auramine staining is a fluorescent staining that requires the examination of the sample under a fluorescence microscope by a parasitologist. The *Cryptosporidium* spp. acid-resistant oocysts are observed as a brilliant green-yellow colour.

### 2.3. Data Collection

Clinical data such as age, sex, immunosuppression, weight loss, diarrhoea, abdominal pain, and clinical attitude after microbiological information were obtained from medical records. Additionally, other epidemiologic data were also recorded, including recent history of international travel and swimming pool, recreational, or tap water exposition.

### 2.4. Statistical Analysis

Data were recorded, and univariate non-stratified analysis was performed using the Stata 14 (StataCorp LP, Lakeway, TX, USA) statistical package.

## 3. Results

During the studied period, we received 21,688 stool samples for parasitological study. A total of 453 (2.08%) were positive for *Cryptosporidium* spp. using the ICT method. These samples belonged to 422 patients, of which 296 (70.14%) cases were confirmed using auramine staining. However, 126 (29.86%) subjects had discrepant results: a positive test using RIDA^®^QUICK ICT but negative auramine staining.

Serial stool samples were available from 43 patients. A total of 30 patients had concordant results of all serial stool samples, and no intra-patient variability was detected. A total of 13 patients had discrepant results: 10/13 patients showed ICT-positive results in all serial stool samples, but not all stool samples were positive for auramine staining, and 3/13 patients showed ICT-positive results in all serial stool samples but auramine-negative staining in all serial stool samples.

Out of 422 patients, 49.29% were females and 50.71% were males. In the group of not confirmed cases, females were predominant, but no statistically significant differences were detected (*p* = 0.14) (Table 1). The median age of the patients with an ICT-positive test independent of the result of the auramine staining was 22 years old (1.5 months–89 years), with an incidence peak in one-year-old children. The group of confirmed cases (patients with positive auramine staining) had an average age of 19 years, whereas the not confirmed cases group had an average age of 30 years (*p* < 0.001) (Table 1).

**Figure 1:**
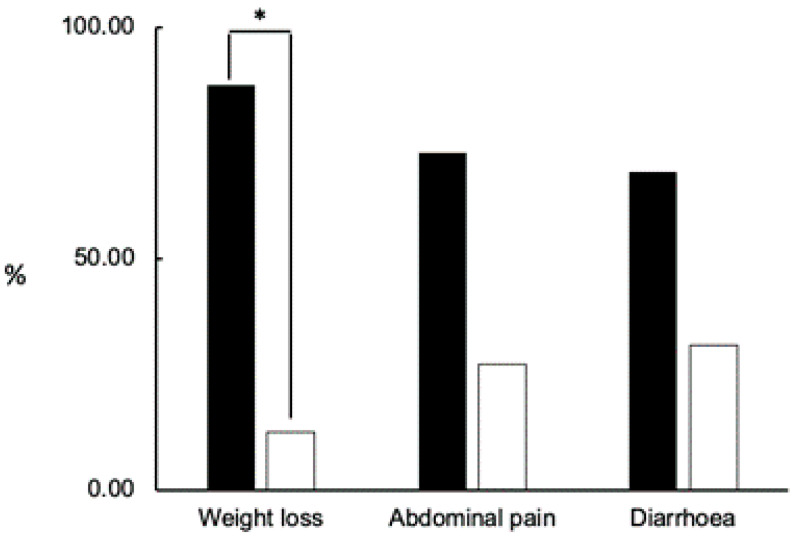
Weight loss was the main symptom related to confirmed cases. Confirmed cases are represented by black bars, and not confirmed cases by white bars. Data correspond to 24 patients who showed weight loss, 59 patients who expressed abdominal pain, and 96 patients with diarrhoea. The statistical significance (*p* < 0.05) was obtained by performing a two-sample *t*-test with equal variances. Differences were considered significant at a two-sided level of *p* < 0.05. * *p* < 0.05.

As we previously commented, *Cryptosporidium* spp. infection can vary from asymptomatic infection to life-threatening illness. Symptoms were present in 112 patients with an ICT positive result and were present in 80% of them independently if they were confirmed or not confirmed cases with no statistically significant differences. In the other 20% of cases, no symptoms related to *Cryptosporidium* spp. infections were described in the clinical history. Weight loss was manifested by 24 of the 112 studied patients, and it was the only symptom that showed statistically significant differences between both groups (*p* = 0.012), being more frequent in confirmed cases (*n* = 21, 87.5%) (Figure 1). Abdominal pain was also present, especially in confirmed cases (*n*= 43, 72.8%), and the difference was almost statistically significant (*p* = 0.064) (Figure 1). In the present study, 114 of 422 patients presented an immunosuppression condition without any significant differences between confirmed and not confirmed groups (*p* = 0.64) (Table 1).

Patients with a confirmed presence of oocysts in faeces had a history of international travel more frequently than the unconfirmed group, showing an almost statistically significant difference (*p* = 0.08) (Table 1). The relation of swimming pool environments with *Cryptosporidium* spp. confirmed cases could not be established because the medical records of studied patients did not show this exposition factor. Regarding drinking tap water, only two patients belonging to unconfirmed groups could be studied, and if anyone showed exposure to this risk factor, then no comparison could be established.

**Figure 2:**
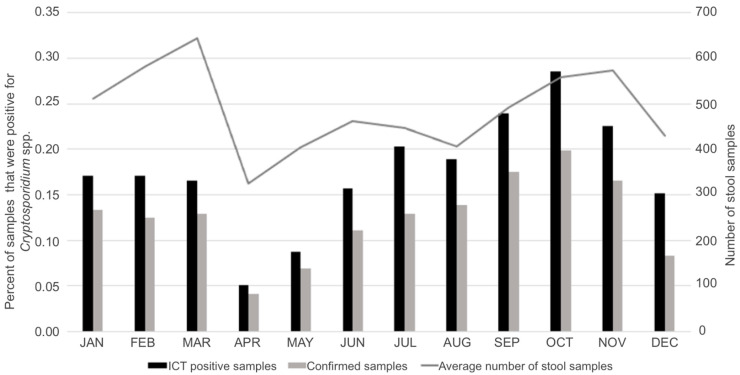
Annual distribution of cryptosporidiosis cases diagnosed in the Microbiology and Parasitology service of the Hospital Universitari Germans Trias i Pujol from 2016 to 2019. Grey line shows the monthly average of stool samples received in the Microbiology and Parasitology service during the studied period.

Regarding seasonality, a similar annual distribution was observed for total ICT-positive samples and those confirmed by auramine staining, with a large peak from July to October, especially in September and October (Figure 2).

Concerning the clinical decision-making after receiving the microbiological results, there was a tendency to treat more patients in whom ICT was confirmed by auramine than in those with discordant results with an almost statistically significant difference (*p* = 0.08) (Table 1).

## 4. Discussion

*Cryptosporidium* spp. is a recognised global burden and a significant cause of morbidity and mortality. Routine parasite tests in stools cannot identify *Cryptosporidium* spp. cases unless specific tests are performed. According to the Surveillance Atlas of Infectious Diseases data from the ECDC, there were 1511 confirmed cases in Spain, with a growing trend since 2014, when 326 cases were reported [13]. In this sense, we observe that during the studied period, the proportion of *Cryptosporidium* spp. cases was around 2% (*n* = 422) in patients from the Metropolitan North Area of Barcelona, Maresme, and Vallés Occidental, demonstrating its circulation in these areas. As we previously commented, some people can be asymptomatic carriers of *Cryptosporidium* spp. Therefore, the real prevalence of cryptosporidiosis could be underestimated because asymptomatic healthy subjects probably do not contact a healthcare provider for parasitological screening. In this line, an epidemiologic study performed in France shows that a systematic search for *Cryptosporidium* spp. DNA in stool samples revealed a higher prevalence of cryptosporidiosis cases in immunocompetent patients than those previously reported because diarrhoea aetiologies are rarely investigated [8]. This could reinforce the importance of screening populations to detect asymptomatic cases, control outbreaks, and perform public health interventions.

Currently, there are no international standard methods for the diagnosis of cryptosporidiosis. Although molecular diagnosis options are increasing, techniques based on microscopy direct observation remain the gold standard for *Cryptosporidium* spp. infection [16]. One limitation of microscopy diagnosis can be found in high-throughput microbiology laboratories where the number of samples received per day can exceed the turnaround time, and performing auramine staining on all samples is not a cost-effective strategy. Therefore, screening methods could decrease the workload. In the present study, we used two complementary strategies to confirm the presence of *Cryptosporidium* spp. oocysts in the faeces of patients, first, with an LFIA, and then positive samples were subjected to auramine staining and posterior microscopy observation. Auramine staining has been considered a low-tech method, relatively economical, sensitive, and easy to perform [17,18]. Thus, it is widely employed to detect *Cryptosporidium* spp. oocysts. Nevertheless, this method presents some limitations, such as fluorescence microscopy being required; sometimes small debris can be confused with *Cryptosporidium* species oocysts; it is a time-consuming method and highly dependent on the expertise of the observer. In our cohort, out of the 422 patients with an ICT-positive test in 70.14% of cases, oocytes were observed using auramine staining. These results could be explained because ICT is more specific and sensitive than microscopy observation [19]. On the one hand, the ICT method achieves a sensitivity of 93%, whereas that achieved with auramine staining varies from 90 to 100% [20,21]. The higher sensitivity of the ICT method compared with auramine staining is also elucidated in the results obtained from serial stool samples. In parasitological tests, serial stool sample collection is extremely important due to the non-continuous excretion of the parasite from the host. In those cases where all serial stool samples were positive for ICT but oocytes were observed with auramine staining, there were probably low loads of oocytes in the faeces. In this line, it has been previously described that microscopy can be false-negative for cases with a low parasite density, especially in the late phase of the infection or when intact oocytes are absent and during intermittent elimination of *Cryptosporidium* spp. oocysts through faeces [22]. On the other hand, one of the limitations of the ICT method is that some false positives have also been found in faeces in the presence of high amounts of erythrocytes, polymorphonuclear cells, and yeast (data not published).

Confirmed patients had an average age of 19 years and were younger than the unconfirmed group. Thus, it is concordant with the fact that cryptosporidiosis especially affects young people [4]. In our hospital attendance area, *Cryptosporidium* spp. infection is the third principal mandatory notifiable causal agent of severe gastroenteritis after rotavirus and *Giardia lamblia* infection [23]. These data invite us to reconsider the confirmation of *Cryptosporidium* species oocysts in the stool of patients under 19 years according to our algorithm.

We observed that symptoms presented by patients with positive ICT independently of the result of auramine staining were weight loss, diarrhoea, and abdominal pain. Nevertheless, the symptoms presented more frequently in the confirmed group were weight loss and abdominal pain. This is unexpected because the main manifestation of cryptosporidiosis is watery diarrhoea [8]. Other concomitant illnesses, especially those that affect the gastrointestinal tract, should be ruled out to confirm the results obtained in the present work. We also note that 20% of patients who are screened for parasites in faeces and showed an ICT-positive result apparently do not have symptoms related to *Cryptosporidium* spp. infection. One plausible explanation is that these subjects were asymptomatic carriers of *Cryptosporidium* spp. and the detection of oocysts in their faeces could be considered a casual finding. It is also possible that the symptomatology presented by these subjects was available in their medical record.

Patients with both positive methods showed a history of international travel more often than the unconfirmed group. This indicates that, in our cohort, international travel could be one of the main risk factors for acquiring *Cryptosporidium* spp. and cryptosporidiosis must be ruled out as a cause of gastroenteritis in patients with travel history. In Spain, the National Network of Epidemiological Surveillance has established some protocols to avoid, as far as possible, the transmission and acquisition of *Cryptosporidium* species oocysts and control outbreaks [23]. Nevertheless, in other regions, especially in developing countries, there are no hygiene measures efficient enough to eliminate harmful microorganisms, promoting their transmission. Some studies have shown that crowded living conditions, animal contact, and open defecation are responsible for the majority of *Cryptosporidium* spp. cases in low- and middle-income countries [24].

Regarding the seasonality of cryptosporidiosis, in the studied area, there is a pronounced seasonal increase in cases occurring in late summer–early autumn, according to previous studies performed in Europe [25]. A meta-analysis of the effect of seasonality showed that high ambient temperature (more important in temperate countries) and high rainfall (more important in the tropics) were associated with an increased risk of cryptosporidiosis [26]. In our area, the detected incidence peak could also be attributed to increased travel and exposure to recreational water, although this information could not be obtained from the clinical history of all studied patients.

Concerning clinical attitude, we found that confirmed cases tend to receive treatment more frequently than unconfirmed cases. As we previously commented, the visualisation of *Cryptosporidium* spp. oocytes under the microscope probably depend on the amount in the original sample. According to this fact, it has been described that symptomatic cases generally contain larger numbers of oocysts than asymptomatic ones [27].

## 5. Conclusions

The two-step diagnosis algorithm could be especially useful in hospitals with a high number of stool samples received for parasitological testing. To speed up the clinical assistance, we use RIDA^®^QUICK ICT to screen the stool samples for *Cryptosporidium* spp. oocytes. As a screening method, ICT is a highly-sensitive technique that could overestimate the results; therefore, positive results must be confirmed using microscopic observation. During the study, it was shown that ICT could have false positives in some samples, and the microscopy visualisation could be negative in faeces with low loads of oocytes. Thus, the authors of this manuscript state that performing the two-step algorithm may improve the cryptosporidiosis diagnosis.

The ICT method established a proportion of 2% of *Cryptosporidium* spp. cases in the HGTiP influence area from 2016 to 2019, demonstrating its circulation in the Metropolitan North Area of Barcelona, Maresme, and Vallés Occidental. These samples belonged to 422 patients, of which 70.14% of cases were confirmed using auramine staining. The ICT results are confirmed, especially in young people and patients with a history of international travel, which seem to be the two main risk factors for acquiring *Cryptosporidium* spp. infection in the studied area. A positive ICT in this epidemiologic group could not necessarily be confirmed using auramine staining and microscope observation, although further studies are needed.

One of the limitations found in the present work is the study of immunodeficient patients. It has been described in other studies that solid organ transplanted patients are more prone to suffer cryptosporidiosis even more than HIV patients [17]. Another limitation we found during the study is the lack of information in some medical reports. As we previously commented, it would be very important to rule out other infections to correlate the symptoms of the patients with the *Cryptosporidium* spp. detection, but we do not have access to that information. In this line, machine learning approaches could allow the classification of clinical data. Then, the next step for improving the epidemiological study of *Cryptosporidium* spp. cases in the HGTiP influence area could be the creation of a form to facilitate appropriate and prospective data collection by doctors, especially those related to exposition factors.

A correct diagnostic algorithm for *Cryptosporidium* spp. detection could prevent and control possible outbreaks produced in the Metropolitan North Area of Barcelona, Maresme, and Vallés Occidental. We conclude that *Cryptosporidium* spp. detection should be included in every microbiology laboratory routine as a cause of gastrointestinal complaints. Few laboratories in our country actively search for *Cryptosporidium* spp. as a diarrhoea etiological agent. With this study, we encourage other laboratories to adapt their diagnosis algorithm and investigate *Cryptosporidium* spp. in their routine as a causative agent of gastrointestinal complaints.

## Figures and Tables

**Table 1:** Characteristics of confirmed and unconfirmed *Cryptosporidium* spp. patients. The first column shows the studied parameters. The second and third columns show the mean age and percentage of patients of confirmed and unconfirmed groups, respectively, which present the characteristics mentioned in the first column. The last column expresses the statistical significance through the *p*-values obtained by performing a two-sample *t*-test with equal variances.

Characteristics	Confirmed Group	Unconfirmed Group	*p* Value
Mean age	19	30	<0.001
Female sex %	46.9	54.7	0.14
Immunosuppression %	3.8	5.7	0.64
International travel %	38.9	19	0.08
Treatment %	53.2	35.2	0.08

## Data Availability

Data are available on reasonable request from the corresponding author.
